# Factor Xa inhibitor, edoxaban ameliorates renal injury after subtotal nephrectomy by reducing epithelial‐mesenchymal transition and inflammatory response

**DOI:** 10.14814/phy2.15218

**Published:** 2022-03-09

**Authors:** Lixin Fang, Koji Ohashi, Hayato Ogawa, Naoya Otaka, Hiroshi Kawanishi, Tomonobu Takikawa, Yuta Ozaki, Kunihiko Takahara, Minako Tatsumi, Mikito Takefuji, Toyoaki Murohara, Noriyuki Ouchi

**Affiliations:** ^1^ Department of Cardiology Nagoya University Graduate School of Medicine Nagoya Japan; ^2^ Department of Molecular Medicine and Cardiology Nagoya University Graduate School of Medicine Nagoya Japan

**Keywords:** Chronic kidney disease

## Abstract

Chronic kidney disease (CKD) is an increasing and life‐threatening disease worldwide. Recent evidence indicates that blood coagulation factors promote renal dysfunction in CKD patients. Activated factor X (FXa) inhibitors are safe and first‐line drugs for the prevention of thrombosis in patients with atrial fibrillation. Here, we investigated the therapeutic effects of edoxaban on CKD using the mouse 5/6 nephrectomy model. Eight‐week‐old wild‐type mice were subjected to 5/6 nephrectomy surgery and randomly assigned to two groups, edoxaban or vehicle admixture diet. Edoxaban treatment led to reduction of urinary albumin excretion and plasma UN levels compared with vehicle group, which was accompanied with reduced glomerular cross‐sectional area and cell number. Edoxaban treatment also attenuated fibrinogen positive area in the remnant kidneys after subtotal nephrectomy. Moreover, edoxaban treatment resulted in attenuated tubulointerstitial fibrosis after 5/6 nephrectomy, which was accompanied by reduced expression levels of epithelial‐mesenchymal transition (EMT) markers, inflammatory mediators, and oxidative stress markers in the remnant kidneys. Treatment of cultured proximal tubular cells, HK‐2 cells, with FXa protein led to increased expression levels of EMT markers, inflammatory mediators, and oxidative stress markers, which were abolished by pretreatment with edoxaban. Treatment of HK‐2 cells with edoxaban attenuated FXa‐stimulated phosphorylation levels of extracellular signal‐regulated kinase (ERK) and NF‐κB. Our findings indicate that edoxaban can improve renal injury after subtotal nephrectomy by reducing EMT and inflammatory response, suggesting that FXa inhibition could be a novel therapeutic target for CKD patients with atrial fibrillation.

## Graphical abstract

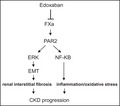

**News and Noteworthy**

Edoxaban treatment ameliorated renal function after subtotal nephrectomy with accompanying increases in EMT markers, inflammatory mediators, and oxidative stress markers. Consistently, treatment of HK‐2 cells with FXa increased EMT markers, inflammatory mediators, and oxidative stress markers, which were abolished by edoxaban treatment. Finally, FXa treatment increased phosphorylation levels of ERK and NF‐κB, which were abolished by edoxaban treatment through PAR2‐dependent mechanisms. Thus, edoxaban can improve renal injury by reducing EMT and inflammatory response.

## 1 INTRODUCTION

According to the increase of aging population, the prevalence of chronic kidney disease (CKD) continues to increase, and CKD patients are estimated to be 8%–16% of the adult population all over the world (Jha et al., 2013). Renal function is declined according to aging even in healthy subjects without causative diseases, such as type 2 diabetes and chronic glomerulonephritis (Imai et al., 2008). Recent evidence shows that CKD patients commonly have blood coagulation disorders, and according to the progression of the CKD stage, blood coagulation factors are increased, thereby leading to further CKD progression (Huang et al., 2017; Wang et al., 1997). However, little is known about the contribution of coagulation factors to the development of CKD.

Activated factor X (FXa) is a key regulator of both intrinsic‐ and extrinsic‐coagulating cascades. FXa and its receptor protease‐activated receptor (PAR) 2 signaling play an important role in various disorders including inflammation, fibrosis, and atherosclerosis (Grandaliano et al., 2003; Hara et al., 2015; Shinagawa et al., 2007; Zuo et al., 2015). FXa inhibitors, such as direct oral anticoagulants (DOACs), are widely used for the prevention of stroke and other thrombotic complications of non‐valvular atrial fibrillation. Recently, Horinouchi et al. (2018) reported that oral administration of edoxaban, which is a FXa inhibitor, ameliorated renal fibrosis in a mouse model of unilateral urinary obstruction. Here, we investigated the therapeutic effects of edoxaban on CKD using a mouse model of 5/6 nephrectomy. Furthermore, we investigated the effects of FXa on fibrosis, inflammatory response, and oxidative stress in cultured proximal tubular epithelial cells.

## 2 MATERIALS AND METHODS

### 2.1 Materials

Edoxaban was kindly provided by Daiichi Sankyo Co., Ltd. Antibodies against phosphorylated NF‐κB p65 (Ser‐536), phosphorylated extracellular signal‐regulated kinase (ERK) (Thr‐202/Tyr‐204), ERK, NF‐κB p65, and α‐tubulin were purchased from Cell Signaling Technology. Recombinant FXa protein was purchased from Haematologic Technologies Inc. U0126 was purchased from Cell Signaling Technology.

### 2.2 Animal and surgical procedure of subtotal nephrectomy

Male wild‐type (WT) mice in a background of C57BL/6J at the age of 8 weeks were subjected to subtotal (5/6) nephrectomy operation, as previously described (Hayakawa et al., 2015; Ohashi et al., 2007). Briefly, the upper and lower poles of the left kidney (two‐thirds of the left kidney) were resected. After 1 week, the remaining right kidney was removed through a right paramedian incision after ligation of the right renal artery, vein, and ureter. Seven days after ablation, WT mice were fed normal diets containing edoxaban (25 mg/kg/day) or vehicle for 7 weeks. Eight weeks after ablation, WT mice were sacrificed for analysis. Before the surgical procedure, anesthetization (medetomidine, midazolam, and butorphanol at doses of 0.15, 2.0, and 2.5 mg/kg, respectively) was administered intraperitoneally. The adequacy of anesthesia was confirmed by the lack of a toe‐pinch withdrawal response during the surgical procedure. Study protocols were approved by the Institutional Animal Care and Use Committee at Nagoya University.

### 2.3 Laboratory methods

At 8 weeks from operation, mice were sacrificed for analysis. Collected blood and urine samples were used for analysis. Plasma concentrations of urea nitrogen (UN) and creatinine (Cr) and urine concentrations of Cr were measured in a commercial laboratory (SRL). Urinary albumin concentration was measured by a murine albumin enzyme‐linked immunosorbent assay (ELISA) kit (Exocell). Urinary albumin excretion was evaluated as albumin/gram of urinary Cr. Plasma FXa concentration was measured by a murine FXa ELISA kit (MyBioSource).

### 2.4 Histological analyses

Tissue samples were fixed by 4% paraformaldehyde and embedded in paraffin. Serial tissue sections (5 μm) of the kidney were stained with hematoxylin and eosin (H‐E) and Masson's trichrome (Sigma). In immunohistochemistry, the kidney tissues were stained with antibodies for fibrinogen (Thermo Fisher Scientific). Intraglomerular cell number, glomerular size, fibrinogen positive area, and fibrosis area were measured by using an ImageJ analysis system (Ohashi et al., 2007).

### 2.5 Cell culture

HK‐2 cells were purchased from the American Type Culture Collection. The cells were cultured in medium consisting of DMEM/F12 (Gibco) supplemented with 10% FBS at intervals of 3–4 days to continuously passaged. HK‐2 cells were placed in DMEM/F12 medium for 16 h and cultured in the presence or absence of edoxaban (100 μmol/L) for 1 h, followed by stimulation with FXa (100 nmol/L) or vehicle for 24 h. In some experiments, HK‐2 cells were treated with vehicle or U0126 (20 μmol/L), which is an ERK inhibitor, for 1 h, followed by stimulation with FXa (100 nmol/L) or vehicle for 24 h. Knockdown of PAR2 was achieved by siRNA transduction at 25 nM with lipofectamine RNAiMAX (Invitrogen) 24 h before experiments. Lipofectamine RNAiMAX and siRNAs were dissolved in Opti‐MEM (Gibco). ON‐TARGETplus siRNA SMART pools targeting PAR2 were purchased from Horizon Discovery. Control cultures were transfected with unrelated scrambled siRNA (ON‐TARGET plus Control Non‐Targeting Pool, Horizon Discovery).

### 2.6 Quantitative real‐time PCR methods

Gene expression levels were quantified by the real‐time PCR method. Total RNA was extracted with RNeasy‐Mini Kit (Qiagen) according to the manufacturer's protocol (Ogura et al., 2012). Extracted RNA was reverse‐transcribed by using the ReverTra Ace (Toyobo). PCR procedure was performed with a Bio‐Rad real‐time PCR detection system using THUNDERBIRD SYBR qPCR Mix as a double‐standard DNA‐specific dye. Primers are listed in Table 1. All results were normalized to 36B4.

**TABLE 1:** Primers used for quantitative RT‐PCR

Mouse	
36B4:	forward 5'‐GCTCCAAGCAGATGCAGCA‐3' reverse 5'‐CCGGATGTGAGGCAGCAG‐3'
Collagen I:	forward 5'‐GTCCCAACCCCCAAAGAC‐3' reverse 5'‐CAGCTTCTGAGTTTGGTGATA‐3'
Collagen III:	forward 5'‐TGGTTTCTTCTCACCCTTCTT‐3’ reverse 5'‐TGCATCCCAATTCATCTACGT‐3'
TGFβ1:	forward 5’‐CACCGGAGAGCCCTGGATA‐3' reverse 5’‐TTCCAACCCAGGTCCTTCCT‐3'
αSMA:	forward 5’‐AAGAGGAAGACAGCACAGCC‐3' reverse 5’‐AGCGTCAGGATCCCTCTCTT‐3'
N‐Cadherin:	forward 5’‐GGGACAGGAACACTGCAAAT‐3' reverse 5’‐CGGTTGATGGTCCAGTTTCT‐3'
Vimentin:	forward 5’‐GGCTGCGAGAGAAATGC‐3' reverse 5’‐CCACTTTCCGTTCAAGGTCAAG‐3'
TNFα:	forward 5’‐ CGGAGTCCGGGCAGGT‐3' reverse 5’‐GCTGGGTAGAGAATGGATGAACA‐3'
MCP1:	forward 5'‐CCACTCACCTGCTGCTACTCAT‐3' reverse 5'‐TGGTGATCCTCTTGTAGCTCTCC‐3'
gp91^phox^:	forward 5'‐TTGGGTCAGCACTGGCTCTG‐3' reverse 5'‐TGGCGGTGTGCAGTGCTATC‐3'
p47^phox^:	forward 5'‐GATGTTCCCCATTGAGGCCG‐3' reverse 5'‐GTTTCAGGTCATCAGGCCGC‐3'
p67^phox^:	forward 5'‐CTGGCTGAGGCCATCAGACT‐3' reverse 5'‐ AGGCCACTGCAGAGTGCTTG‐3'

Human	
36B4:	forward 5'‐TGCTCAACATCTCCCCCTTCTC‐3' reverse 5'‐ACCAAATCCCATATCCTCGTCC‐3'
αSMA:	forward 5'‐CCGTGATCTCCTTCTGCATT‐3' reverse 5'‐CTGTTCCAGCCATCCTTCAT‐3'
N‐Cadherin:	forward 5'‐TGTTTGACTATGAAGGCAGTGG‐3' reverse 5'‐TCAGTCATCACCTCCACCAT‐3'
Vimentin:	forward 5'‐TGCTTCTCTGGCACGTCTTG‐3' reverse 5'‐GGACATGCTGTTCCTGAATCTG‐3'
IL6:	forward 5'‐CTTCCATCCAGTTGCCTTCTTG‐3' reverse 5'‐AATTAAGCCTCCGACTTGTGAAG‐3'
TNFα:	forward 5'‐AACCTCCTCTCTGCCATCAA‐3' reverse 5'‐ATGTTCGTCCTCCTCACAGG‐3'
MCP1:	forward 5'‐CGACATCCTGGAACTGCCCTACC‐3' reverse 5'‐CACTGTGCCGCTCTCGTTCAC‐3'
gp91^phox^:	forward 5'‐TAGTGGGAGCAGGGATTG‐3' reverse 5'‐TCAAAGGCATGTGTGTCC‐3'
p22^phox^:	forward 5'‐ATTGTGGCGGGCGTGTT‐3' reverse 5'‐GCACCGAGAGCAGGAGAT‐3'
p47^phox^:	forward 5'‐CCTGACGAGACGGAAGACC‐3' reverse 5'‐CTTTCCTGATGACCCACCA‐3'
PAR2:	forward 5'‐CTGCATCTGTCCTCACTGGA‐3' reverse 5'‐ACAGAGAGGAGGTCAGCCAA‐3'

Abbreviations: TGFβ1; transforming growth factor β1, αSMA; α‐smooth muscle actin, TNFα; tumor necrosis factor α, MCP1; monocyte chemotactic protein 1, PAR2; protease‐activated receptor 2, IL6; interleukin 6.

### 2.7 Western blot analysis

Tissue or cell samples were homogenized in lysis buffer (Cell Signaling Technology) containing 1mM PMSF (Sigma). Equal amounts of plasma were separated by denaturing SDS‐PAGE and transferred onto PVDF membranes. Membranes were incubated with the primary antibodies, followed by incubation with the HRP‐conjugated secondary antibodies. ECL prime system (GE Healthcare) was used for the detection of the protein signal. The protein expression levels were determined by measurement of the band intensities using ImageJ software (National Institute of Health, USA) (Schneider et al., 2012).

### 2.8 Statistical analysis

Data are presented as mean ± S.E.M. The differences between two groups for variables with normal distributions were evaluated by unpaired Student's *t*‐test. Differences between three or more groups were evaluated using one‐way analysis of variance (ANOVA), with a post hoc Tukey's test. The differences between groups for variables with non‐normal distribution were analyzed by the Steel‐Dwass test (for three or more groups). Data distributions were evaluated by Shapiro–Wilk test. *p *< 0.05 denoted the presence of a statistically significant difference. All statistical analyses were performed using JMP Pro 15 software (SAS).

## 3 RESULTS

### 3.1 Edoxaban attenuates renal damage and intraglomerular fibrin deposition after subtotal nephrectomy

**FIGURE 1:**
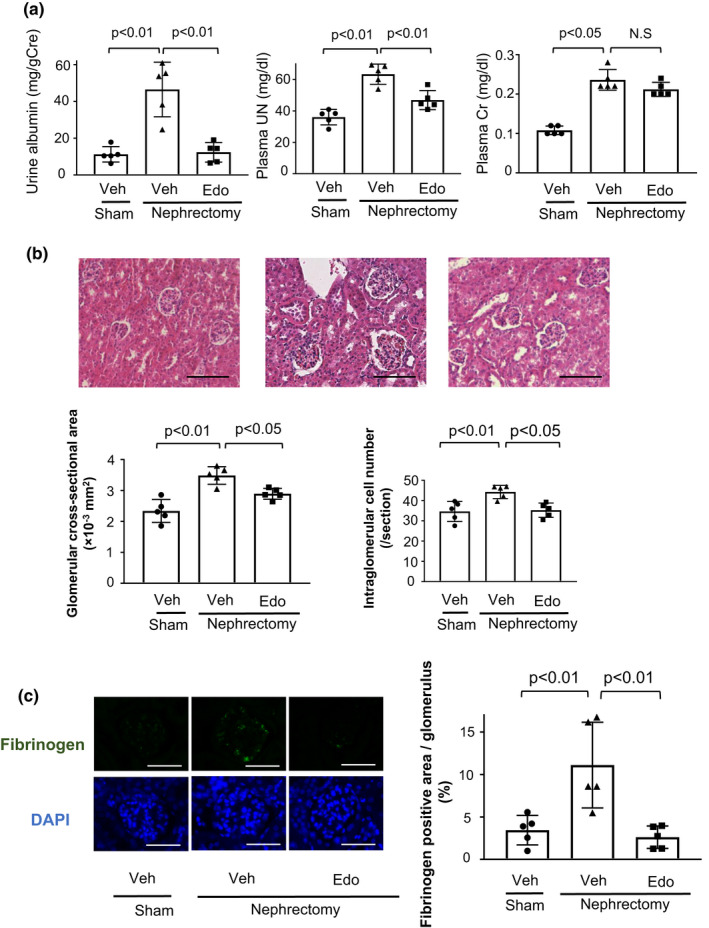
Edoxaban attenuates renal damage and intraglomerular fibrin deposition after 5/6 nephrectomy. (a) Urine and plasma parameters of renal function in wild‐type (WT) mice fed normal diets containing edoxaban (Edo) or vehicle (Veh) after subtotal nephrectomy or sham operation. The left panel shows urinary albumin excretion normalized to urinary Cr. Center and right panels show plasma concentration of UN and Cr of vehicle‐treated (Veh) and edoxaban‐treated WT mice (Edo) after subtotal nephrectomy or sham operation; *N *= 5 in each group. (b) Histological analysis of glomerular hypertrophy. Upper panels show representative photos of the kidneys from vehicle‐treated mice (Veh) and edoxaban‐treated mice (Edo) after subtotal nephrectomy or sham operation. Lower panels show quantitative analyses of glomerular cross‐sectional area (left) and intraglomerular cell number (right) as measured by ImageJ. *N* = 5 in each group. Scale bars show 100 μm. (c) Immunohistochemical staining against fibrinogen and DAPI. The left panels show representative photos of the kidneys from vehicle‐treated mice (Veh) and edoxaban‐treated mice (Edo) after subtotal nephrectomy or sham operation. The right panel shows quantitative analyses of fibrinogen positive area in the glomerulus of the kidneys from vehicle‐treated mice (Veh) and edoxaban‐treated mice (Edo) after subtotal nephrectomy or sham operation. *N* = 5 in each group. Scale bars show 50μm. One‐way ANOVA with Tukey's multiple comparisons test (a (urine albumin and plasma UN), b and c), and Steel‐Dwass test (a (plasma Cr)) were used to produce the *p* values. N.S, not significant

To examine the effects of edoxaban on renal injury, wild‐type (WT) mice at 8 weeks of age were subjected to 5/6 nephrectomy and randomly assigned to two groups, normal diet or edoxaban admixture diet at day 7 after operation. After 7 weeks, mice were sacrificed for analyses after the collection of urine and blood samples. Subtotal nephrectomy significantly increased plasma FXa levels in WT mice (Figure S1). Plasma FXa levels were remarkably reduced in edoxaban (Edo)‐treated WT mice after subtotal nephrectomy compared with vehicle (veh)‐treated WT mice (Figure S1). Subtotal renal ablation significantly increased urinary albumin excretion and circulating levels of urea nitrogen (UN) and creatinine (Cr) levels in WT mice (Figure 1a). Urinary albumin excretion and plasma UN levels were reduced in edoxaban‐treated WT mice after subtotal nephrectomy compared with vehicle‐treated WT mice (Figure 1a). In contrast, plasma Cr levels after renal injury did not significantly differ between edoxaban‐treated and vehicle‐treated WT mice.

To assess glomerular damage after subtotal nephrectomy or sham operation, kidneys of WT mice were stained with Hematoxylin and Eosin (H‐E). Edoxaban‐treated WT mice exhibited reduced glomerular cross‐sectional area and intraglomerular cell number after subtotal nephrectomy compared with vehicle‐treated WT mice (Figure 1b).

To evaluate the effect of edoxaban on intraglomerular microembolism after subtotal nephrectomy or sham operation, kidney tissues of WT mice were immunochemically stained with anti‐fibrinogen antibody. Intraglomerular microembolism shown as fibrin deposition was increased after subtotal nephrectomy operation compared with sham operation, whereas edoxaban treatment significantly reduced intraglomerular microembolism after subtotal nephrectomy compared with vehicle treatment (Figure 1c).

### 3.2 Edoxaban ameliorates tubulointerstitial fibrosis by reducing EMT, inflammatory response, and oxidative stress

**FIGURE 2:**
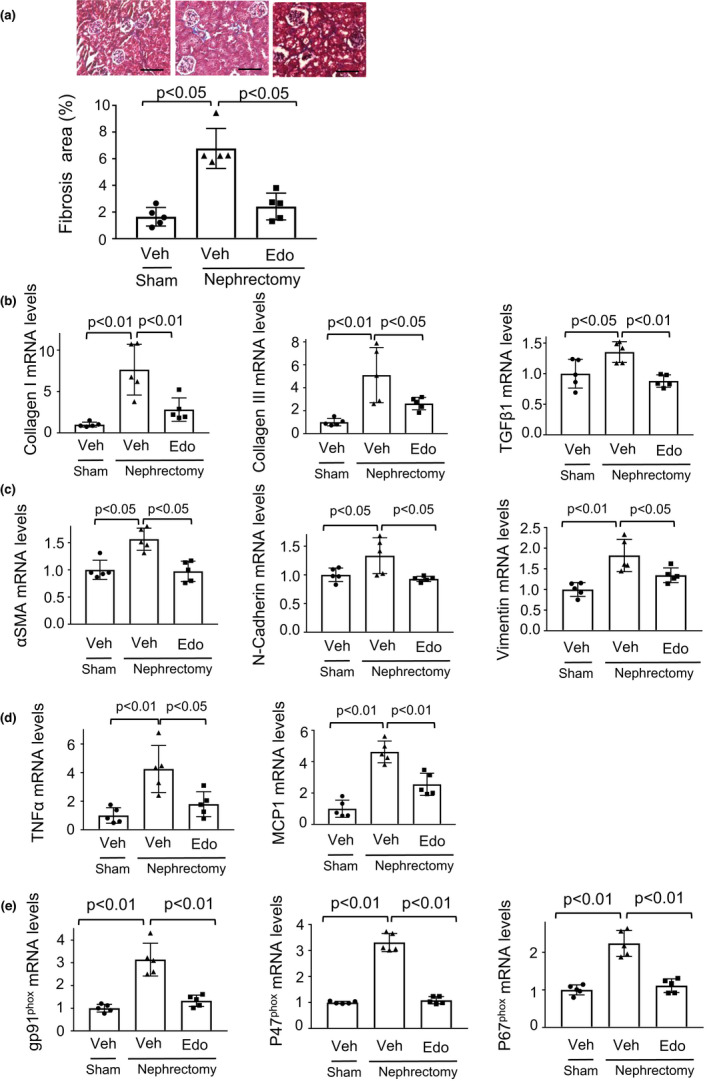
Edoxaban reduces renal tubulointerstitial fibrosis, epithelial‐mesenchymal transition (EMT), inflammatory response, and oxidative stress after 5/6 nephrectomy. (a) Histological evaluation of interstitial fibrosis. Upper panels show representative photos of the kidneys from vehicle‐treated mice (Veh) and edoxaban‐treated mice (Edo) after subtotal nephrectomy or sham operation as determined by Masson trichrome staining. The lower panel shows quantitative analysis of fibrosis area as measured by ImageJ. *N* = 5 in each group. Scale bars show 100 μm. (a) mRNA levels of fibrosis‐associated factors such as collagen I, collagen III, and transforming growth factor (TGF) β1 in the kidney from vehicle‐treated mice (Veh) and edoxaban‐treated mice (Edo) after subtotal nephrectomy or sham operation. *N *= 5 in each group. (c) mRNA levels of EMT markers such as α‐smooth muscle actin (SMA), N‐cadherin, and vimentin in the kidney from normal vehicle‐treated (Veh) and edoxaban‐treated mice (Edo) after subtotal nephrectomy or sham operation. *N* = 5 in each group. (d) mRNA levels of proinflammatory mediators such as TNFα and MCP1 in the kidney from vehicle‐treated mice (Veh) and edoxaban‐treated mice (Edo) after subtotal nephrectomy or sham operation. *N* = 5 in each group. (e) mRNA levels of oxidative stress markers such as gp91^phox^, p47^phox^, and p67^phox^ in the kidney from vehicle‐treated mice (Veh) and edoxaban‐treated mice (Edo) after subtotal nephrectomy or sham operation. *N *= 5 in each group. One‐way ANOVA with Tukey's multiple comparisons test (b, c (N‐cadherin and vimentin), d and e) and Steel‐Dwass test (a and c (α SMA)) were used to produce the *p* values. N.S, not significant

To evaluate interstitial fibrosis after subtotal nephrectomy or sham operation, the kidneys of mice were stained with Masson trichrome. Subtotal nephrectomy increased renal fibrosis area of WT mice, and edoxaban treatment reduced fibrosis area in the injured kidneys of WT mice after subtotal renal ablation compared with vehicle treatment (Figure 2a). Consistently, edoxaban‐treated WT mice had reduced expression levels of fibrosis markers including collagen I, collagen III, and transforming growth factor (TGF) β1, in the remnant kidney after subtotal nephrectomy compared with vehicle‐treated WT mice (Figure 2b). Edoxaban‐treated WT mice also had reduced expression levels of EMT markers including α‐smooth muscle actin (SMA), N‐cadherin, and vimentin in the remnant kidney after subtotal nephrectomy compared with vehicle‐treated WT mice (Figure 2c). Furthermore, edoxaban‐treated WT mice exhibited reduced expression levels of inflammatory mediators including tumor necrosis factor (TNF) α and monocyte chemoattractant protein (MCP) 1, and oxidative stress markers including gp91^phox^, p47^phox^, and p67^phox^ in the remnant kidney after subtotal nephrectomy compared with vehicle‐treated WT mice (Figure 2d,e).

### 3.3 Edoxaban attenuates FXA‐induced increase in EMT, inflammatory response, and oxidative stress in cultured renal tubular epithelial cells

**FIGURE 3:**
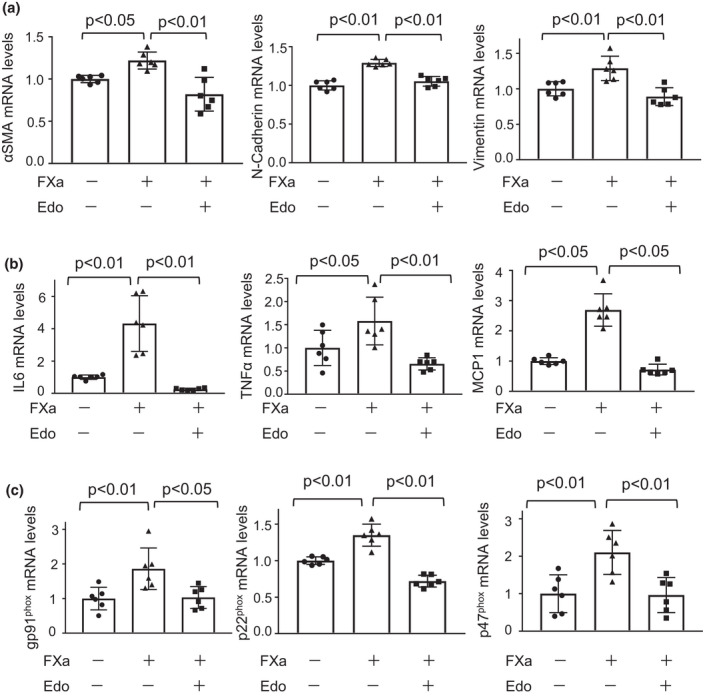
Edoxaban attenuates FXa‐stimulated increase in epithelial‐mesenchymal transition (EMT), inflammatory response, and oxidative stress in HK‐2 cells. a–c) Effect of edoxaban on FXa‐stimulated expression of EMT markers, proinflammatory mediators, and oxidative stress markers in human proximal tubular cell lines, HK‐2 cells. HK‐2 cells were pretreated with edoxaban (Edo) (100 μmol/L) or vehicle for 1 h, followed by incubation in the presence or absence of FXa (100 nmol/L) for 24 h. A, mRNA levels of EMT markers such as α‐smooth muscle actin (SMA), N‐cadherin, and vimentin in HK‐2 cells. B, mRNA levels of proinflammatory mediators such as interleukin (IL) 6, TNFα, and MCP1 in HK‐2 cells. C, mRNA levels of oxidative stress markers such as gp91^phox^, p22^phox^, and p47^phox^ in HK‐2 cells. *N* = 6 in each group. One‐way ANOVA with Tukey's multiple comparisons test (a, b (IL 6 and TNFα) and c) and Steel‐Dwass test (b (MCP1)) were used to produce the *p* values. N.S, not significant

To dissect the precise mechanism by which edoxaban reduced EMT, inflammatory response, and oxidative stress in the remnant kidney after subtotal nephrectomy, human renal proximal tubular epithelial cell line, HK‐2 cell were pretrated with edoxaban or vehicle, followed by FXa treatment. Pretreatment of HK‐2 cells with edoxaban significantly reduced FXa‐stimulated expression of EMT markers including α‐SMA, N‐cadherin, and vimentin (Figure 3a). Furthermore, pretreatment of HK‐2 cells with edoxaban significantly attenuated FXa‐stimulated expression of inflammatory mediators, such as interleukin (IL) 6, TNFα, and MCP1 and oxidative stress markers including gp91^phox^, p22^phox^, and p47^phox^ (Figure 3b,c). These results indicate that edoxaban abolished FXa‐stimulated increase in EMT, inflammatory response, and oxidative stress in proximal renal tubular cells.

**FIGURE 4:**
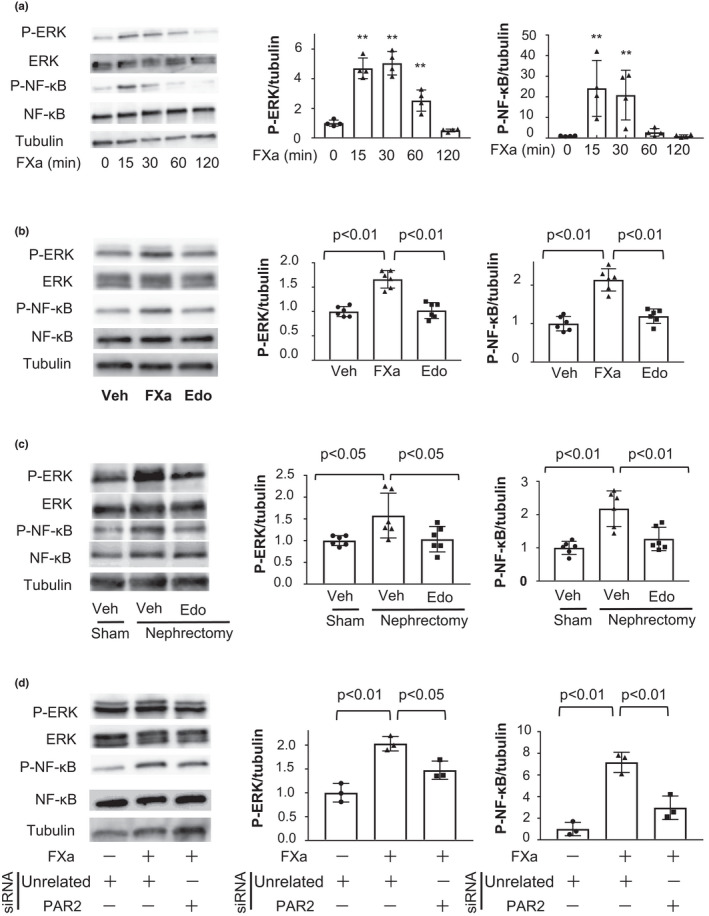
Edoxaban suppresses FXa‐stimulated phosphorylation of ERK and NF‐κB in HK‐2 cells. (a) Time‐dependent changes in phosphorylation of ERK and NF‐κB in HK‐2 cells after treatment with FXa (100 nmol/L). The left panel shows representative western blots of phosphorylation of ERK (P‐ERK), ERK, phosphorylation of NF‐κB (P‐NF‐κB), NF‐κB, and α‐tubulin (Tubulin). The center and right panels show the quantitative analysis of P‐ERK/Tubulin and P‐NF‐κB/Tubulin, respectively. *N* = 4 in each group. **p < 0.01 versus 0 min. (b) Effect of edoxaban on FXa‐stimulated increase in phosphorylation levels of ERK and NF‐κB in HK‐2 cells. HK‐2 cells were pretreated with edoxaban (Edo) or vehicle (Veh) for 1 h, followed by FXa treatment for 15 min. The left panel shows representative western blots of P‐ERK, ERK, P‐NF‐κB, NF‐κB, and Tubulin. The center and right panels show quantitative analyses of P‐ERK/Tubulin and P‐NF‐κB/Tubulin. *N *= 6 in each group. (c) Phosphorylation of ERK and NF‐κB in the kidneys of vehicle‐treated WT mice (Veh) or edoxaban‐treated WT mice (Edo) after nephrectomy or sham operation. The left panel shows representative western blots of P‐ERK, ERK, P‐ NF‐κB, NF‐κB, and Tubulin. The center and right panels show quantitative analyses of P‐ERK/Tubulin and P‐NF‐κB/Tubulin. *N* = 6 in each group. (d) HK‐2 cells were transfected with siRNA targeting PAR2 (25 nM) or unrelated siRNA (25 nM) for 24 h, followed by FXa treatment for 15 min. The left panel shows representative western blots of P‐ERK, ERK, P‐ NF‐κB, NF‐κB, and Tubulin. The center and right panels show quantitative analyses of P‐ERK/Tubulin and P‐NF‐κB/Tubulin. *N *= 3 in each group. Student's *t*‐test (a) and One‐way ANOVA with Tukey's multiple comparisons test (b, c and d) were used to produce the *p* values

Because EMT and inflammatory response in proximal tubular epithelial cells is mediated by ERK and NF‐κB signaling, respectively (Hu et al., 2020; Pollack et al., 2007), we assessed the effects of FXa on phosphorylation of ERK and NF‐κB in HK‐2 cells. Treatment of HK‐2 cells with FXa time dependently increased the phosphorylation levels of ERK and NF‐κB (Figure 4a). Pretreatment of HK‐2 cells with edoxaban abolished FXa‐stimulated increase in phosphorylation levels of ERK and NF‐κB (Figure 4b). Consistently, subtotal nephrectomy increased renal phosphorylation levels of ERK and NF‐κB in WT mice, and edoxaban treatment reduced phosphorylation levels of ERK and NF‐κB in the remnant kidney of WT mice after subtotal renal ablation compared with vehicle treatment (Figure 4c). In addition, pretreatment of HK‐2 cells with the ERK inhibitor, U0126 significantly attenuated FXa‐stimulated expression of EMT markers including α‐SMA, N‐cadherin, and vimentin (Figure S2).

Finally, to investigate the contribution of PAR2 to FXa‐stimulated phosphorylation of ERK and NF‐κB, HK‐2 cells were transfected with siRNA targeting PAR2 or unrelated siRNA. Transduction of HK‐2 cells with siRNA targeting PAR2 reduced mRNA expression of PAR2 by 71.4 ± 0.7%. Treatment of HK‐2 cells with siRNA targeting PAR2 diminished FXa‐stimulated phosphorylation of ERK and NF‐κB (Figure 4d). These results suggest that FXa induces ERK and NF‐κB activation through the PAR2‐dependent pathway.

## 4 DISCUSSION

In the present study, our major findings are following: (1) Oral administration of edoxaban ameliorated albuminuria, glomerular hypertrophy, tubulointerstitial fibrosis after subtotal nephrectomy with accompanying increases in expression of EMT markers, inflammatory cytokines, and oxidative stress markers, (2) treatment of cultured human proximal tubular cells, HK‐2 cells, with recombinant FXa protein led to increased expression levels of EMT markers, inflammatory mediators, and oxidative stress markers, which were abolished by edoxaban treatment. (3) Treatment of HK‐2 cells with FXa protein resulted in increased phosphorylation levels of ERK and NF‐κB, which were abolished by edoxaban treatment through PAR2‐dependent mechanisms.

Renal fibrosis, characterized by tubulointerstitial fibrosis, causes tubular atrophy and glomerulosclerosis, thereby leading to disruption of normal kidney function. Residual fibroblasts are mesenchymal cells and function to maintain the structure of organs. On the other hand, myofibroblasts appear dedifferentiated from residual fibroblasts, podocytes, and renal tubular epithelial cells and contribute to pathological fibrosis during the process of CKD progression (Loeffler & Wolf, 2015; Zeisberg & Neilson, 2010). The dedifferentiation from tubular epithelial cells to myofibroblasts, so‐called epithelial‐mesenchymal transition (EMT), has been reported to associate with CKD progression in many animal CKD models (Holian et al., 2008; Lan, 2003; Shimizu et al., 2006; Zeisberg et al., 2008). TGF‐β is the most representative inducer of EMT in many organs (Pardali et al., 2017; Xu et al., 2009). TGF‐β promotes EMT of renal tubular epithelial cells and podocytes, thereby leading to renal fibrosis during CKD progression (Li et al., 2008; Liu, 2004). It has been reported that thrombin induces EMT and collagen production in retinal pigment epithelial cells (Bastiaans et al., 2013). On the other hand, the contribution of coagulation factors to renal fibrosis via EMT has not been elucidated. In the present study, we exhibited for the first time that FXa promotes EMT in HK‐2 cells. Our data suggest that edoxaban suppresses FXa‐induced EMT in proximal tubular cells, thereby leading to reduction of renal interstitial fibrosis.

ERK signaling is reported to mediate EMT in several pathological conditions including diabetic nephropathy (Zhang et al., 2019). In the present study, we found that enhanced ERK phosphorylation was observed in the remnant kidney at 8 weeks after 5/6 nephrectomy operation. A previous report showed that ERK phosphorylation is upregulated even at 12 weeks after 5/6 nephrectomy (Ding et al., 2015). Thus, it is plausible that subtotal nephrectomy induces continuous activation of ERK in the remnant kidney, thereby leading to the development of renal fibrosis, although further investigation will be required to elucidate the precise role of ERK in CKD progression.

CKD patients show higher circulating levels of inflammatory mediators, including TNFα and IL6 (Kitada et al., 2011; Upadhyay et al., 2011; Vinuesa et al., 2006). Proinflammatory cytokines, such as TNFα, promote renal injury and dysfunction, which are accompanied by increased expression of reactive oxygen species (ROS) (Bertani et al., 1989; Navarro‐Gonzalez & Mora‐Fernandez, 2008). In the present study, the expression of inflammatory mediators and oxidative stress markers is increased in the remnant kidney after subtotal nephrectomy compared with the kidney in sham‐operated mice, whereas edoxaban administration reduced inflammatory response and oxidative stress in the remnant kidney after nephrectomy. Furthermore, FXa treatment increased the expression of inflammatory mediators and oxidative stress markers in cultured renal tubular cells. Edoxaban abolished the increased expression of inflammatory mediators and oxidative stress markers stimulated by FXa treatment. Therefore, it is likely that edoxaban can attenuate kidney injury after subtotal nephrectomy partly through the reduction of inflammatory response and oxidative stress.

Coagulation factors promote tissue injury through PARs (PAR1‐4). FXa cleaves the N terminus of PAR2 and augments EMT and inflammation through MAPK or NF‐κB signaling, respectively (Rothmeier & Ruf, 2012). PAR2 is abundantly expressed in the kidney and contributes to the progression of many kidney diseases, such as diabetic nephropathy, crescentic glomerulonephritis, and IgA nephropathy (Grandaliano et al., 2003; Madhusudhan et al., 2016; Moussa et al., 2007; Oe et al., 2016). In the present study, we found that FXa increased phosphorylation levels of NF‐κB and ERK in cultured renal tubular epithelial cells through the PAR2‐dependent mechanism. Edoxaban blocked FXa‐induced increase in EMT, inflammatory response, and oxidative stress as well as NF‐κB and ERK activation in renal tubular epithelial cells. Of note, edoxaban diminished phosphorylation of ERK and NF‐κB in the remnant kidney after subtotal renal ablation. Thus, it is plausible that edoxaban can mitigate renal injury by antagonizing the ability of FXa to enhance renal interstitial fibrosis, inflammation, and oxidative stress through PAR2/ERK or PAR2/ NF‐κB signaling (Figure S3).

In conclusion, we have shown that FXa inhibitor can ameliorate renal function in subtotal nephrectomy model, which is accompanied with reducing tubulointerstitial fibrosis and expression of inflammatory mediators and oxidative stress markers. Thus, FXa inhibition could be a novel therapeutic target for CKD patients with atrial fibrillation.

### 4.1 Translational significance and limitation

We used only one type of CKD model in this study. Although the mouse model of 5/6 nephrectomy is widely used for the study of CKD, this model in C57BL/6 mice does not develop progressive renal dysfunction (Leelahavanichkul et al., 2010). Our data indicate that edoxaban reduced urinary albumin excretion in C57BL/6 mice after 5/6 nephrectomy without affecting plasma Cr levels, suggesting that this may be due to the use of mild renal dysfunction model. Further investigation will be required to clarify the effect of edoxaban on severer renal dysfunction using models of advanced CKD including glomerulonephritis and lupus nephritis.

In the present study, we found the renoprotective effects of edoxaban using a model of mild renal dysfunction. Because direct oral anti‐coagulant drugs including edoxaban are prohibited to use among patients with end‐stage renal disease, our findings indicate that edoxaban could be beneficial for the early‐stage CKD in patients with atrial fibrillation.

## CONFLICT OF INTEREST

The authors have declared that no conflict of interest exists.

## ETHICS STATEMENT

Study protocols of animals were approved by the Institutional Animal Care and Use Committee at Nagoya University.

## AUTHOR CONTRIBUTIONS

LF designed the research study, conducted the experiments, acquired the data, analyzed the data, and wrote the manuscript. KO designed the research study, conducted the experiments, acquired the data, analyzed the data, provided expertise related to the experiments, and wrote the manuscript. HO, NO (N. Otaka), HK, TT, YO, KT, and MT (M. Tatsumi) conducted the experiments, acquired the data, and analyzed the data. MT (M. Takefuji) and TM designed the research study and provided expertise related to the experiments. NO (N. Ouchi) designed the research study, analyzed the data, provided expertise related to the experiments, and wrote the manuscript.

## Supporting information



Supplementary Material
