# Supplementary material for: Factor Xa inhibitor, edoxaban ameliorates renal injury after subtotal nephrectomy by reducing epithelial‐mesenchymal transition and inflammatory response
Source: Physiol Rep. 2022 Mar 9;10(5):e15218. doi: 10.14814/phy2.15218 (PMC8905573; doi:10.14814/phy2.15218)

Supplementary data to:

Factor Xa inhibitor, edoxaban ameliorates renal injury after subtotal nephrectomy by reducing epithelial-mesenchymal transition and inflammatory response

Lixin Fang[1], Koji Ohashi[2], Hayato Ogawa[1], Naoya Otaka[1], Hiroshi Kawanishi[1], Tomonobu Takikawa[1], Yuta Ozaki[1], Kunihiko Takahara[1], Minako Tatsumi[2], Mikito Takefuji[1], Toyoaki Murohara[1] and Noriyuki Ouchi[2]

[1] Department of Cardiology, Nagoya University Graduate School of Medicine, Nagoya, Japan

[2] Department of Molecular Medicine and Cardiology, Nagoya University Graduate School of Medicine, Nagoya, Japan

Address correspondence to:

Koji Ohashi, MD, PhD. or Noriyuki Ouchi, MD, PhD.

Department of Molecular Medicine and Cardiology

Nagoya University Graduate School of Medicine

65 Tsurumai-cho, Showa-ku, Nagoya, 466-8550, Japan

Tel: +81-52-744-2427

Fax: +81-52-744-2427

E-mail: ohashik@med.nagoya-u.ac.jp or nouchi@med.nagoya-u.ac.jp

Supplemental Figure Legend

Supplemental Figure 1. Plasma FXa levels in vehicle-treated (Veh) and edoxaban-treated (Edo) WT mice at 8 weeks after subtotal nephrectomy or sham operation. N=5 in each group. One-way ANOVA with Tukey's multiple comparisons test was used to produce the P values.

Supplemental Figure 2. Effect of the ERK inhibitor U0126 on FXa-stimulated expression of epithelial mesenchymal transition (EMT) markers in HK-2 cells. HK-2 cells were pretreated with U0126 (20 μmol/L) or vehicle for 1 h followed by stimulation with FXa (100 nmol/L) or vehicle for 24 h. mRNA levels of EMT markers such as α-smooth muscle actin (SMA), N-cadherin and vimentin were determined by real-time PCR methods. N=6 in each group. One-way ANOVA with Tukey's multiple comparisons test was used to produce the P values.

Supplemental Figure 3. Proposed scheme of the possible mechanisms by which edoxaban and activated factor X (FXa) modulate renal function in chronic kidney disease (CKD). Edoxaban can improve CKD progression by antagonizing the ability of FXa to enhance epithelial mesenchymal transition (EMT)-mediated renal interstitial fibrosis, inflammation and oxidative stress through PAR2/ERK or PAR2/NF-κB signaling.

Supplemental Figure 1

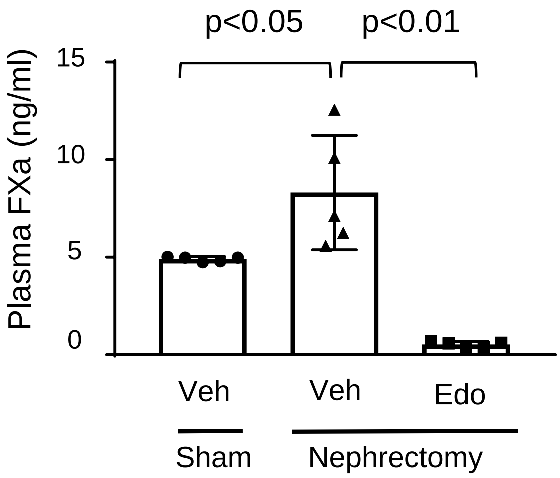

Supplemental Figure 2

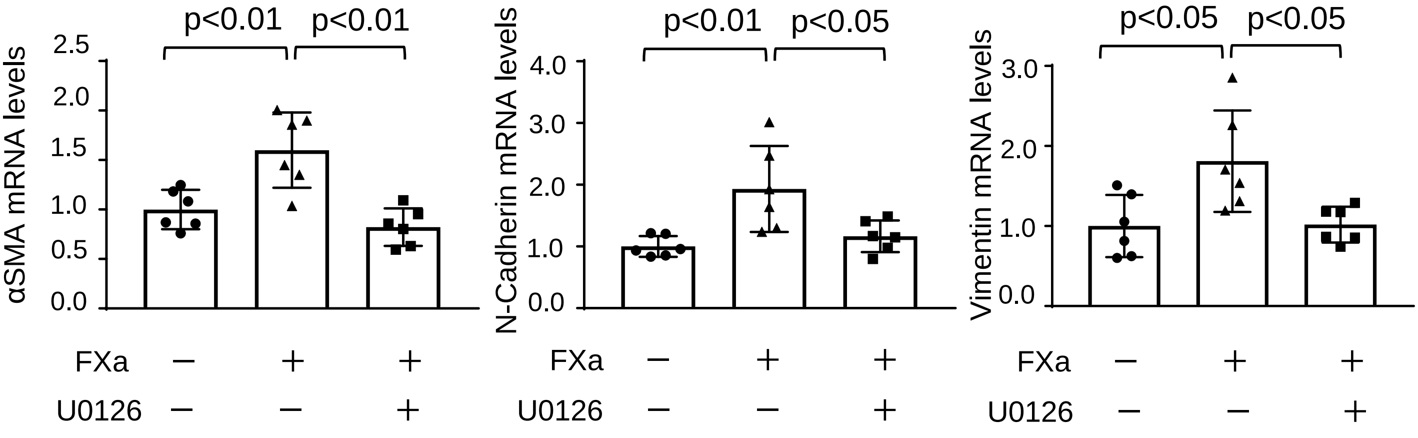

Supplemental Figure 3

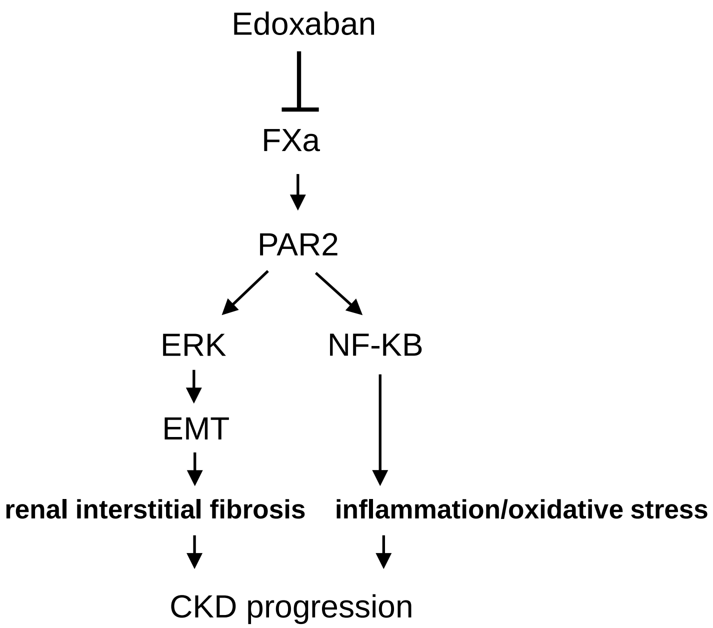

Supplement: Supplementary file 1 — Supplementary Material [file PHY2-10-e15218-s001.pdf]
